# Prioritizing educational initiatives on emerging technologies for Italian pediatricians: bibliometric review and a survey

**DOI:** 10.1186/s13052-023-01512-w

**Published:** 2023-09-05

**Authors:** Alberto Eugenio Tozzi, Francesco Gesualdo, Elisabetta Pandolfi, Diana Ferro, Giulia Cinelli, Elena Bozzola, Tommaso Aversa, Antonio Di Mauro, Chiara Mameli, Ileana Croci

**Affiliations:** 1 Predictive and Preventive Medicine Research Unit, Bambino Gesù Children’s Hospital IRCCS, Rome, Italy; 2 Pediatric Unit, Bambino Gesù Children’s Hospital, IRCCS, Rome, Italy; 3 Department of Human Pathology of Adulthood and Childhood “G. Barresi”, University of Messina, Messina, Italy; 4 Pediatric Unit, University Hospital “G. Martino”, Messina, Italy; 5 University of Bari “Aldo Moro”, Bari, Italy; 6 Department of Pediatrics, V. Buzzi Children’s Hospital, Milan, Italy; 7 Department of Biomedical and Clinical Sciences, University of Milan, Milan, Italy

**Keywords:** Technology, Medical education, Pediatrics, Artificial intelligence, Italy

## Abstract

**Background:**

Emerging technologies have demonstrated outstanding potential in improving healthcare, yet their full integration remains a challenge for all medical specialties, including pediatrics. To support the swift implementation of technologies, we identified the current trends through a bibliometric review, and we conducted a survey on Italian pediatricians to gauge educational needs and willingness to integrate technologies into clinical practice.

**Methods:**

A working group of pediatricians representing various backgrounds designed and coordinated the study. To identify relevant topics for educational strategy development, we focused on virtual reality, telehealth, natural language processing, smartphone applications, robotics, genomics, and artificial intelligence. A bibliometric analysis limited to 2018–2023 was performed to identify trends and emerging applications within each topic. Based on the results, a questionnaire was developed and made available online to all Italian pediatricians. The results were analyzed through descriptive analysis and a multivariable logistic regression to explore associations between technology adoption and sociodemographic characteristics.

**Results:**

A total of 3,253 publications were found, with Telehealth and Telemedicine having the highest number of publications and Natural Language Processing the lowest. The number of respondents to the online questionnaire was 1,540, predominantly medical doctors with over 20 years of experience working as family pediatricians. Telehealth had the highest level of knowledge (95.2%), followed by smartphone applications (89.1%) and genomics (63.2%). The greatest potential for increased use through education programs was projected for natural language processing (+ 43.1%), artificial intelligence (+ 39.6%), and virtual and mixed reality (+ 38.1%). Female respondents and older individuals were less likely to use emerging technologies. Hospital pediatricians and residents were more likely to use AI.

**Conclusions:**

We developed a replicable strategy to identify emerging themes in medical technologies relevant to pediatrics and assess the educational needs of pediatricians. A significant gap still exists between current and potential usage of emerging technologies among Italian pediatricians although they showed a positive attitude towards implementing these technologies following specific education programs. The study highlights the need for comprehensive education programs on emerging technologies in pediatrics and recommends addressing gender and age disparities in technology adoption.

**Supplementary Information:**

The online version contains supplementary material available at 10.1186/s13052-023-01512-w.

## Introduction

Emerging technologies for healthcare have shown great potential in improving clinical practice, with growing evidence supporting their positive impact [1–4]. However, the full integration of these technologies into healthcare systems is still a distant reality. One of the primary barriers to the transformation of healthcare driven by these technologies is the inadequate education provided to healthcare workers during their medical training [5, 6]. Despite some reviews highlighting the advantages of digital education for healthcare professionals in general and specifically in pediatrics [7, 8], there is currently a lack of sufficient educational initiatives in this field. As a result, healthcare workers often lack the necessary competency and comfort with the utilization of these technologies [9–12].

Education programs focused on emerging technologies for medical applications should incorporate key components such as digital literacy, a profound understanding of genomics, and comprehensive training in conducting traditional clinical procedures using cutting-edge tools like telehealth, virtual reality, and artificial intelligence-based devices. By equipping healthcare professionals with these skills, they can optimize workflows, enhance the quality of care, and improve patients’ overall comfort and well-being [5].

Digital technologies are gradually finding their way into care pathways for both adult medicine and pediatrics [13–15]. However, it is essential to note that technologies developed for adults cannot be directly applied to pediatrics. There are several reasons for this distinction. Firstly, investing in prevention has the most significant impact on children, and therefore, technologies should be focused on achieving this objective. Secondly, the rapid and dynamic nature of children’s biology and growth necessitates continuous adaptation of technologies to ensure optimal health outcomes. Lastly, the epidemiology of most pediatric diseases significantly differs from that of adults, which demands technologies to be specifically tailored to address these unique aspects.

While robust evidence on the impact of many emerging technologies is still being gathered, several recent studies have explored their applications in pediatrics. Among these technologies, telehealth and telemonitoring have demonstrated their utility in preventing the spread of infections, such as during the COVID-19 pandemic, while providing essential healthcare services [16, 17]. Virtual reality (VR) is also increasingly recognized as a valuable tool for non-pharmacological pain control in pediatric patients [18]. Robotics has emerged as a valuable resource for rehabilitation in various pediatric conditions [19]. Genomics is progressively being integrated into clinical pathways, particularly for rare diseases, as part of the pursuit of precision medicine [20]. Additionally, artificial intelligence (AI) shows excellent promise in almost all domains of pediatrics and it has already proven to be a powerful tool in diagnostic image interpretation, particularly in cases of pediatric brain tumors [21].

To support the swift implementation of emerging technologies in pediatrics, it is crucial to have a well-defined strategy for educating pediatricians. While certain frameworks for developing technology education in healthcare workers have been proposed [22], they often struggle to keep up with the rapid changes and advancements in this field. Therefore, we have developed a strategy aimed at promptly informing the curricula of pediatricians with the latest knowledge on emerging technologies, which can be regularly updated. This study presents the outcomes of our strategy, tailored explicitly to Italian pediatricians. The approach encompasses two main components: (1) Identifying the current trends in emerging technologies in pediatrics based on scientific literature; (2) Surveying Italian pediatricians to gauge their educational needs and determine their willingness to integrate these trending topics into their clinical practice.

## Methods

### Board of experts

To ensure a comprehensive perspective, we formed a working group of six pediatricians with diverse backgrounds and roles, representing family practitioners, hospital-based specialists, and academic professionals. The primary aim of this functional focus group was to collectively determine an effective strategy for gathering data to inform educational initiatives for pediatricians regarding emerging technologies. Throughout the study, this group played a pivotal role in coordinating the activities and decision-making processes. They held regular meetings and conducted voting sessions to select the most suitable study procedures and methodologies.

### Bibliometric analysis

To identify the most appropriate topics for developing an education strategy, we began by referring to a prominent review by Topol [5], which suggested several emerging technologies that have the potential to enhance the efficiency and quality of healthcare. These technologies were deemed crucial for the education of the healthcare workforce in the NHS UK. Among the proposed topics in this review, our expert board focused on the following areas: VR, mixed reality (MR), and augmented reality (AR); Telehealth and telemedicine; Natural Language Processing (NLP); Smartphone applications; Robotics; Genomics; and AI.

To gather relevant literature on these topics, specifically within the field of pediatrics, we constructed search queries for each topic. We then utilized the Web of Science database, which provides access to different scientific literature databases to harvest the materials. We limited the search results to publications issued within the past five years and applied the “Pediatrics” filter [Supplementary materials - Appendix 1]. The resulting datasets for each topic were then analyzed using the R software and the Biblioshiny app [23]. This open-source tool for quantitative research facilitates bibliometric analyses, allowing the identification of scientometrics and bibliometrics over time. To gain insight into the emerging applications of these technologies within each topic, we examined those trends using customized field-specific keywords over time. Additionally, we manually classified the publications obtained from this search to quantify the number of clinical trials reported.

### Survey development

The expert board extensively reviewed and discussed the findings from the bibliometric analysis. Based on the results, a preliminary questionnaire was developed, which incorporated inquiries regarding specific applications identified in the bibliometric study.

To refine and finalize the questionnaire, three consecutive iterations were conducted using a Delphi evaluation approach with the expert board. This iterative process allowed for consensus-building and ensured the questionnaire’s content validity. Additionally, a pre-testing phase was carried out with an out-sample group of 20 pediatricians who were not involved in the survey. Their feedback helped refine the questionnaire’s structure and wording, ensuring its clarity and comprehensibility. The items in the finalized questionnaire aimed to investigate various aspects, including the participants’ knowledge, prior experience, and willingness to receive specific education on the emerging topics identified in the bibliometric analysis. Furthermore, the questionnaire explored the likelihood of participants translating the acquired knowledge from these educational activities into clinical practice [Supplementary materials - Appendix 2].

### Survey study design, sample size, and analysis

This cross-sectional study utilized an online questionnaire distributed to the population of Italian pediatricians. The eligible population included all registered pediatricians in Italy, totaling approximately 20,000 individuals. We stratified the sample size by geographic area (northern, central, and southern Italy). The sample size for each geographic area was calculated using an expected proportion of the outcome found in each question of the study at 50% with an accepted margin of error of 5% and a confidence level of 95%, yielding a sample of 377 individuals. Thus, the total sample size for the three geographical areas was estimated at 1131.

To administer the questionnaire, we utilized the SurveyMonkey platform (San Mateo, CA, USA); participation in the survey was voluntary, anonymous, and advertisements were disseminated through various communication channels of major Italian scientific pediatric societies, including newsletters and social media announcements. The survey was available for completion from February 13th to May 2nd, 2023. The target population of Italian pediatricians was not subjected to any specific selection criteria. The collection of responses ceased after obtaining a sufficient number of participants.

Following a descriptive analysis of the collected data, a multivariable logistic regression was conducted to explore associations between the likelihood of using the technology after education and sociodemographic characteristics and items in each of the domains.

In order to address missing data, multiple imputations with chained equations [24] were employed. This method allowed us to generate values for variables with missing data, such as age, professional category (coded as a family pediatrician, hospital pediatrician, university pediatrician, resident, and other), years of experience (coded as less than 5y, 5-10y, 10-20y and more than 20y) and the items in each of the domain (coded as no, yes). All variables included in the predictive models were used to generate imputed values for missing data [24, 25]. It was assumed that the missing data occurred randomly [25]. It is important to note that the outcome variable was not imputed. Data analysis was performed using Stata 17 software (Stata Corporation, College Station, TX, USA).

As no patients were included in this study, a formal protocol was not submitted to an ethical committee. However, we notified the study to the Ethical Committee of the Bambino Gesù Children’s Hospital.

## Results

### Bibliometric analysis

After conducting the search for articles, a total of 3,253 publications were found. Among the different topics, Telehealth and Telemedicine had the highest number of publications, while Natural Language Processing had the lowest. The distribution of keywords with an increasing trend within each topic is outlined in Table 1.

**Table 1:** Keywords emerging from the bibliometric analysis in the seven topics explored

Virtual, mixed and augmented reality (total publications = 230 N. of RCT = 52)		Natural Language Processing (total publications = 108 N. of RCT = 0)		Smartphone Applications (total publications = 329 N. of RCT = 40)		Robotics (total publications = 247 N. of RCT = 8)	
**keyword**	**freq.**	**keyword**	**freq.**	**keyword**	**freq.**	**keyword**	**freq.**
Pain	31	Hearing loss	48	Neuropsychiatric disorders	21	Rehabilitation	10
Neuropsychiatric disorders	31	Speech	21	Chronic diseases	21	Social support	4
Medical education	17			Health promotion	19	Palliative care	1
Anxiety	16			Therapies adherence	8	Social robots	1
**Genomics** **(total publications = 822** ** N. of RCT = 7)**		**Telehealth and telemedicine (total publications = 955** ** N. of RCT = 39)**		**Artificial Intelligence (total publications = 562** ** N. of RCT = 8)**			
**keyword**	**freq.**	**keyword**	**freq.**	**keyword**	**freq.**		
Prognosis	122	Covid-19	281	Prognosis	115		
Syndrome	48	Monitoring	215	Prediction	42		
Rare diseases	25	Neuropsychiatric disorders	68	Covid-19	16		
Diagnosis	23	Emergency	29	Diagnosis	16		
		Education	17	Personalized medicine	9		

The most frequent emerging keyword was COVID-19 in the Telehealth topic, as expected. However, for other topics, we observed a relatively low frequency of specific keywords. Specifically, in the Robotics topic, although there was a scarcity of keywords reporting applications related to social support and palliative care, the Board of experts recommended considering these areas. Specific keywords that emerged from the bibliometric analysis were deemed too narrow for the topic and were consequently excluded. They included: balance, anesthesia, surgery, computer tomography, radiology, and exoskeleton.

The number of clinical trials among the publications included in the analysis varied from 22.6 to 0% for natural language processing, reflecting the maturity and diffusion of specific technologies.

### Pediatricians’ survey

A total of 1,650 pediatricians accessed the online questionnaire. Among them, 32 individuals did not consent to participate, and 78 did not fully complete the questionnaire. Consequently, we included 1,540 responses for analysis. The general characteristics of the respondents are outlined in Table 2.

**Table 2:** Characteristics of the study population (n = 1540)

	n	%
Female	1062	69.1
Age (years)		
Mean (SD)	56.7 (11.0)	
Median (range)	60 (25–84)	
Geographic area		
Northern Italy	695	45.1
Central Italy	401	26.0
Southern Italy	444	28.8
Professional category		
Family pediatrician	1157	76.4
Hospital pediatrician	201	13.3
University pediatrician	14	0.9
Resident	47	3.1
Other	96	6.3
Years of experience		
< 5y	270	18.3
5-10y	130	8.8
10-20y	233	15.8
>20y	841	57.1

The majority of respondents consisted of medical doctors with more than 20 years of experience serving as family pediatricians.

**Fig. 1:**
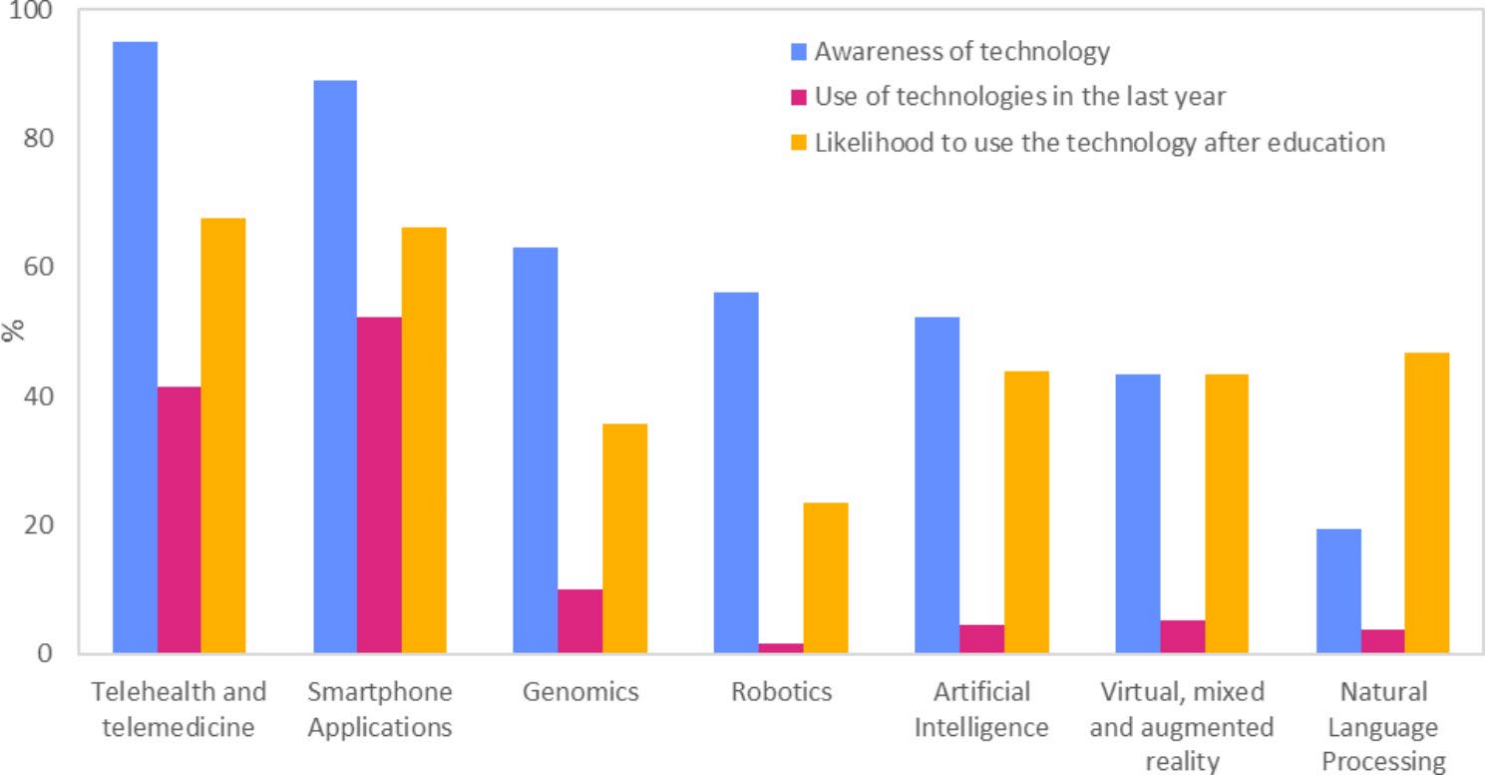
Comparison of awareness, current use, and potential use of emerging technologies in the seven domains included in the study

In Fig. 1, we compared the awareness, recent use, and potential use of emerging technologies across the seven topics examined in this study.

Among the topics, telehealth received the highest level of knowledge, followed by smartphone applications and genomics. However, the recent utilization of these technologies was relatively limited across most categories, with smartphone apps being the most widely used (more than 50%) and telehealth being used by nearly 40% of respondents. The most significant potential increase in technology use, resulting from specific education programs, was projected for NLP (+ 43%), AI (+ 39%), and VR and MR (+ 38%). With education programs, the anticipated future adoption of these technologies would be as high as 70% for telehealth and smartphone apps, and nearly 40% for AI, NLP, and VR and MR.

**Fig. 2:**
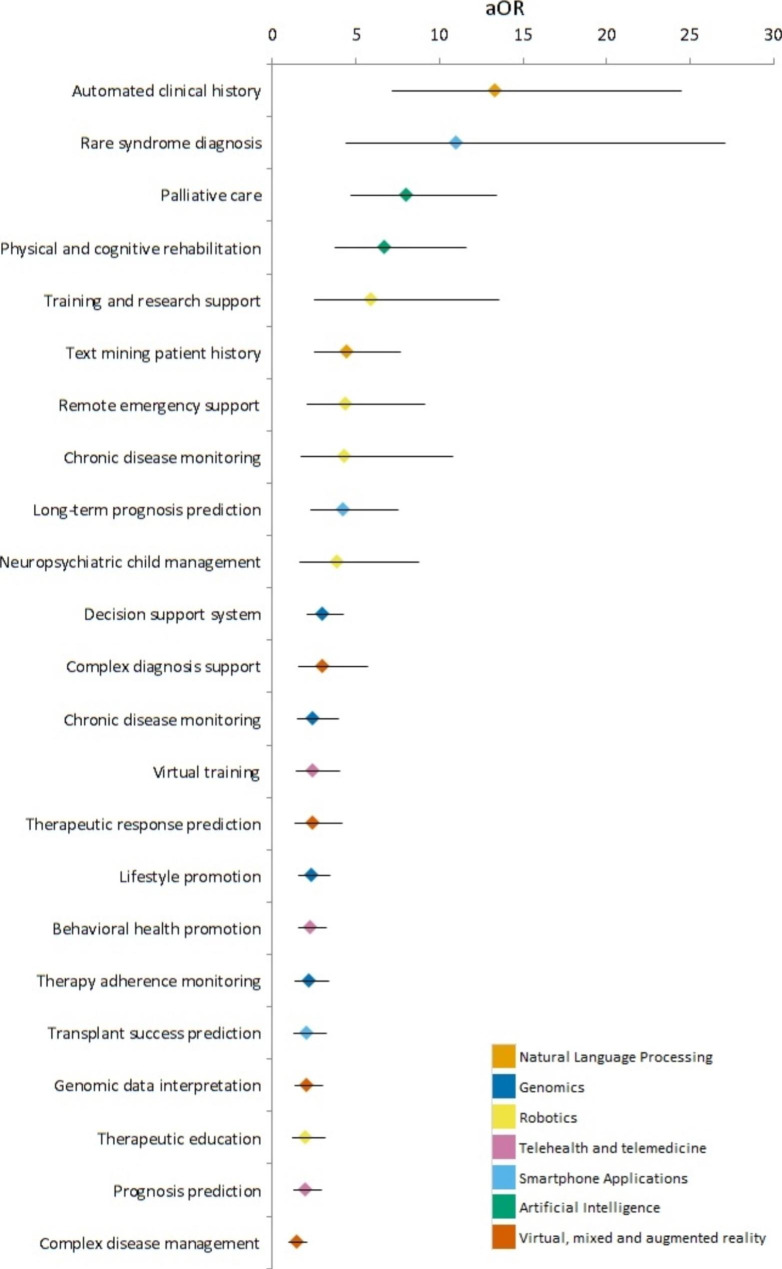
Adjusted odd ratios of implementation of a technology associated with education interest in education in specific areas

Figure 2 presents the associations between the interest in receiving education for a specific application and the likelihood of using that technology in routine clinical practice. These association measures indicate the topics where education programs would yield the highest.

Among the respondents, those who expressed the highest interest in automatically recording the patient and family’s medical history in the electronic medical record during conversations were significantly more likely to use natural language processing in their clinical practice (adjusted odds ratio [aOR] = 13.29 [95%confidence interval [CI]: 7.20-24.52]). Similarly, those who expressed interest in using genomics for diagnosing rare diseases were also significantly more likely to incorporate this technology into their clinical practice (aOR = 10.94 [95%CI: 4.41–27.12]).

In terms of demographic factors, female respondents were less likely to use VR and MR technologies even after attending specific education activities (aOR = 0.63 [95%CI: 0.48–0.82]). They were also less likely to use telemedicine and telehealth technologies (aOR = 0.30 [95%CI: 0.13–0.69]), smartphone apps (aOR = 0.58 [95%CI: 0.42–0.81]), genomics (aOR = 0.44 [95%CI: 0.28–0.69]), and AI (aOR = 0.53 [95%CI: 0.39–0.72]).

Age also influenced technology usage, with younger pediatricians being more likely to use NLP (aOR = 0.96 [95%CI: 0.93–0.99]), telemedicine (aOR = 0.91 [95%CI:0.86–0.96]), and smartphone apps (aOR = 0.97 [95%CI: 0.95–0.99]). Hospital pediatricians, on the other hand, were less likely to use NLP (aOR = 0.41 [0.95%CI: 0.21–0.80]), but more likely to use AI (aOR = 1.73 [95%CI: 1.10–2.71]), as well as residents (aOR = 4.39 [95%CI: 1.72–11.21]). Additionally, pediatricians with more extensive experience were more likely to use NLP (aOR = 3.28 [95%CI: 1.25–8.61] for 5–10 years of experience and aOR = 3.31 [95%CI: 1.38–7.95] for 10–20 years of experience) and telemedicine/telehealth (aOR = 3.63 [95%CI: 1.07–12.35] for 10–20 years of experience and aOR = 3.54 [95%CI: 1.00-12.52] for more than 20 years of experience).

## Discussion

We have developed a replicable and straightforward strategy to identify emerging themes in medical technologies relevant to pediatrics and assess the educational needs of Italian pediatricians in this field. Our approach not only considered the perceived needs of pediatricians based on emerging themes in the literature but also prioritized educational programs based on the likelihood of implementing these technologies in clinical practice.

Through bibliometric analysis, we identified telemedicine and COVID-19, genomic prediction of prognosis, and artificial intelligence as the most trending topics in the literature. Pediatric telehealth has gained significant attention in the medical literature during the pandemic, and its relevant technologies have been increasingly refined and integrated into the pediatric patient journey, regardless of the need to limit social contact [26, 27]. The widespread interest in telehealth during the pandemic has also highlighted the importance of healthcare professionals’ competency in its implementation [28, 29]. Genomics and artificial intelligence are expected to play a crucial role in predicting the prognosis of severe diseases, particularly cancer, and supporting clinical decision-making [30, 31]. Mental health in children has gained increased attention during the pandemic, leading to proposed solutions utilizing various technologies [32, 33].

Our investigation of the education needs and the likelihood of implementing these technologies among Italian pediatricians revealed a significant gap between current and potential usage. However, respondents had a strong positive attitude towards implementing these technologies following specific education programs. We found that the greatest interest in education was related to NLP, with nearly 50% of respondents considering it a potential routine tool in clinical practice. Specifically, the ability to use NLP to automatically generate a clinical history from conversations between pediatricians and families was identified as the most anticipated application. The growing interest in NLP applications has spurred further research in this area [34], and technology companies have already announced the availability of tools for this purpose [35, 36].

AI was identified as a technology with the potential to become a practical tool in the clinic for over 40% of respondents. Its application was particularly anticipated in supporting complex diagnoses and predicting the response to therapeutic interventions. Personalized medicine represents a goal for pediatricians who may feel uncertain when making decisions regarding the most appropriate disease management, especially when faced with complex clinical problems within the constraints of limited time and resources. Technology, including AI, can serve as a powerful tool in addressing these complexities [37].

In contrast, genomics and robotics were considered less likely to be implemented in the clinic, although there was recognition that investing in education for genomics, specifically for the diagnosis of rare syndromes and conditions, could yield valuable returns. These findings were expected, given that the majority of respondents worked in primary care settings.

Our results also revealed that being male and younger were associated with a higher likelihood of implementing emerging technologies in clinical routines. It is well-known that a gender bias exists in the utilization of technology [38] and that young generations are more familiar with technology for socio-cultural reasons. Additionally, Italy still has room for improvement in digital competence compared to other European countries, as indicated by the Digital Economy and Society Index, which places Italy lower than most other countries [39]. These observations have important implications for developing and implementing modern education programs, which should aim to address and balance these inequalities.

Currently, medical education does not adequately prepare pediatricians to leverage the benefits of emerging technologies fully [40]. While courses on emerging technologies exist for healthcare professionals [12], they are not integrated into medical curricula and are generally not specific to pediatrics. Furthermore, the existing educational programs on technology vary significantly and require standardization [22]. Adapting general courses may not be suitable for pediatrics, considering the unique biology and growth of the pediatric population. In contrast, our strategy was designed for pediatrics and can be easily updated according to specific trends. While waiting for deeper integration of knowledge on emerging technologies into medical curricula [11], the development of rapid education programs based on emerging trends in the literature and educational needs represents a feasible strategy for pediatrics, particularly considering the short life cycle of these technologies, which may be better addressed with complementary programs [41].

A systematic review of digital education in pediatrics has demonstrated that several post-registration programs available in various regions of the world are at least as effective as traditional learning methods [8]. This observation supports the consideration of developing online courses for the rapid dissemination of the findings from this study.

Developing effective courses on emerging technologies in pediatric care presents several challenges. Given the rapid pace at which technology evolves, long-lasting learning programs are crucial to keep pediatric healthcare professionals up-to-date with the latest developments. The field of emerging technologies also demands competences from various disciplines which underlines the importance of multi- and cross-disciplinarity in education programs [5]. Moreover, there is a need for a dedicated effort to train new instructors in this field and courses on emerging technologies should be co-designed with pediatricians to ensure that the content is tailored to meet the specific needs and challenges of pediatric care. Effective courses should encompass not only the technical aspects of emerging technologies but also foster literacy about health data sources and governance. Finally, ethical and legal implications should be an integral part of education programs on emerging technologies [5].

Our work has some limitations. To select the main topics for investigating emerging technologies, we took advantage of a widely recognized review that identified the most disruptive technologies for healthcare, expected to significantly improve efficiency and quality of care [5]. It is unlikely that trending topics on emerging technologies were overlooked in this research, as the topics covered in the review were broad and comprehensive.

We employed a bibliometric approach to identify trending topics, regardless of the available evidence, which would have required a systematic review of the literature. This approach is valuable in identifying the most discussed topics suitable for educational programs, where the available evidence regarding efficacy, accuracy, and safety can be reviewed during education. Of note, our bibliometric analysis showed a variable proportion of randomized clinical trials across different topics. As evidence supporting these emerging technologies is expected to evolve rapidly, education programs will be helpful to address this issue and recommend translating the available evidence into practice.

Participation in our survey was voluntary, and it is possible that respondents with a higher interest in technologies were more likely to participate, potentially biasing the results towards positive responses. This factor should be considered when interpreting the findings. On the other hand, the median age of the respondents was high, reflecting the age distribution of the general target population, which may be less inclined to adopt emerging technologies.

## Conclusions

The results of this study have provided insights into the priorities for pediatric education, which will inform the development of educational programs on emerging technologies for Italian pediatricians. Specifically, the use of NLP, AI and VR in the clinic may increase with an educational support. We recommend to repeat this strategy periodically to account for the rapid changes in potential applications of these technologies.

### Electronic supplementary material

Below is the link to the electronic supplementary material.

Supplementary Material 1

Supplementary Material 2

## Data Availability

The datasets during and/or analyzed during the current study available from the corresponding author on reasonable request.

## Abbreviations

VR: virtual reality
MR: mixed reality
AR: augmented reality
AI: artificial intelligence
NHS: National Health System
NLP: natural language processing
aOR: adjusted Odds Ratio
CI: confidence interval
