# Supplementary material for: Prioritizing educational initiatives on emerging technologies for Italian pediatricians: bibliometric review and a survey
Source: Ital J Pediatr. 2023 Sep 5;49:112. doi: 10.1186/s13052-023-01512-w (PMC10478260; doi:10.1186/s13052-023-01512-w)
Supplement: Supplementary file 2 — Supplementary Material 2 [file 13052_2023_1512_MOESM2_ESM.docx]

*Appendix 1*

**Search string (number of articles extracted)**

Virtual, mixed and augmented reality (n=230):

Virtual reality OR augmented reality OR mixed reality

Telehealth and telemedicine (n=955):

Telehealth OR telemedicine

Natural Language Processing (n=108):

speech recognition* OR nlp OR natural language processing

Smartphone Applications (n=329):

app

Robotics (n=247):

robotic*

Genomics (n=822):

genome

Artificial Intelligence (n=562):

artificial intelligence OR machine learning OR deep learning

*Appendix 2*

**Questionnaire**

**Questionnaire on training needs in terms of technology for pediatrics**

**General Information Section**

1) Sex:

a. Male

b. Female

2) Age

3) In which region do you work?

4) In which province do you work?

5) Which category of medical profession do you belong to?

a. Family pediatrician

b. Hospital pediatrician

c. University pediatrician

d. Resident

e. Other

6) How many years have you been working in your current role?

a. Less than 5 years

b. From 5 to 10 years

c. From 10 to 20 years

d. More than 20 years

**Emerging technologies section**

**Virtual, mixed and augmented reality**

*These are technologies which use headsets to immerse oneself in a three-dimensional environment. In virtual reality applications, the environment is closed and is completely separated from the external one. In augmented and mixed reality there is an interaction with the external environment.*

7) In the last year have you ever used Virtual, Augmented or Mixed Reality in your clinical activity?

8) Are you aware of the possibility of using Virtual, Augmented or Mixed Reality technologies in

clinical practice?

9) For which kinds of applications would you like to increase your knowledge of Virtual, Augmented or

Mixed Reality? *(more than one answer possible)*

a. The non-pharmacological treatment of pain and anxiety

b. The treatment of neuropsychiatric diseases

c. Training doctors through the simulation of clinical scenarios

d. Health promoting behavioral therapies

e. The integration of outdoor physical activity

f. To support therapeutic education in children and adolescents with chronic pathologies (e.g.

Type 1 diabetes)

10) Once you gain more expertise in this area, how likely are you to use it in clinical practice?

a. Not at all/unlikely

b. Neutral

c. Very/highly probable

**Interpretation and synthesis of written and spoken natural language**

*A technology based on artificial intelligence that allows you to analyze and interpret written or spoken texts. The same type of technology can be used to synthesize the human voice.*

11) In the last year, have you ever used technologies based on the Recognition and Interpretation of

Natural Language in your clinical activity?

12) Are you aware of the possibility of using Natural Language Recognition and Interpretation

technologies in clinical practice?

13) For which specialized applications would you like to increase your knowledge on Natural

Language Recognition and Interpretation? *(more than one answer possible)*

a. Voice to text assistance for patients with deafness

b. Speech synthesis in the patient with dysarthria

c. Searching and extracting synthetic information from the text of the patient history

d. Automatic recording of the anamnesis in the electronic medical record during the

conversation with the patient and family

14) Once you gain more expertise in this area, how likely are you to use it in clinical practice?

a. Not at all/unlikely

b. Neutral

c. Very/highly probable

**Telemedicine**

*A range of technologies for remote patient monitoring and assistance. It includes technologies for wearable and movable sensors, telecommunication and digital communication.*

15) In the last year have you ever used technologies based on Telemedicine in your clinical activity?

16) Are you aware of the possibility of using Telemedicine technologies in clinical practice?

17) For which specialized applications would you like to increase your knowledge of Telemedicine?

*(more than one answer possible)*

a. Remote monitoring of patients with COVID-19

b. Monitoring of patients with chronic disease

c. Screening for retinopathy in newborns

d. Management of children with neuropsychiatric problems

e. Remote rehabilitation

f. Remote emergency support

g. Support for training and scientific research activities

18) Once you gain more expertise in this area, how likely are you to use it in clinical practice?

a. Not at all/unlikely

b. Neutral

c. Very/highly probable

**Smartphone applications**

*Various technologies characterized by the possibility of use via smartphones. They can be used by patients to periodically receive personalized recommendations, and by doctors to communicate with patients or to consult reference information.*

19) In the last year have you ever used or had your patients use smartphone applications?

20) Are you aware of the possibility of using smartphone applications in clinical practice?

21) For which specialized applications would you like to increase your knowledge of smartphone

applications? *(more than one answer possible)*

a. The promotion of healthy lifestyles

b. Monitoring chronic diseases

c. Monitoring adherence to therapies

d. Monitoring jaundice in newborns

e. Treatment and rehabilitation of certain pathologies such as neuropsychiatric conditions

f. For clinical decision support system

22) Once you gain more expertise in this area, how likely are you to use it in clinical practice?

a. Not at all/unlikely

b. Neutral

c. Very/highly probable

**Robotics**

*A discipline in which engineering, mathematical and artificial intelligence skills converge. Robotics is rapidly developing for clinical applications in which automation of some processes or remote-controlled actions is required.*

23) In the last year, have you ever used technologies based on robotics in your clinical activity?

24) Are you aware of the possibility of using robotic technologies in clinical practice?

25) For which specialized applications would you like to increase your robotics knowledge? *(more*

*than one answer possible)*

a. Physical and cognitive rehabilitation

b. Social support in stressful conditions such as in palliative care

26) Once you gain more expertise in this area, how likely are you to use it in clinical practice?

a. Not at all/unlikely

b. Neutral

c. Very/highly probable

**Genomics**

*A discipline that studies the genome and its evolution. Given the complexity of the data and results addressed, this discipline often makes use of bioinformatics skills.*

27) In the last year, have you ever used technologies based on genomics?

28) Are you aware of the possibility of taking advantage of the information arising from genomics in

clinical practice?

29) For which specialized applications would you like to increase your knowledge of genomics? *(more*

*than one answer possible)*

a. Diagnosis of rare syndromes and diseases

b. Prediction of long-term prognosis and the risks of developing chronic noncommunicable

diseases

c. Prediction of success in organ or stem cell transplants

30) Once you gain more expertise in this area, how likely are you to use it in clinical practice?

a. Not at all/unlikely

b. Neutral

c. Very/highly probable

**Artificial intelligence**

*A technology that performs some actions similar to human behavior. In the clinical environment it has great prospects in the field of precision medicine development.*

31) In the last year, have you ever used technologies based on artificial intelligence in your clinical

activity?

32) Are you aware of the possibility of using artificial intelligence technologies in clinical practice?

33) For which specialized applications would you like to increase your knowledge of artificial

intelligence? *(more than one answer possible)*

a. Support in complex diagnoses

b. The interpretation of diagnostic images

c. Predicting disease prognosis

d. The interpretation of genomic and metagenomic data

e.The classification of diseases

f. The creation of personalized therapies

g. Prediction of responses to given therapeutic interventions

h. Prediction of adverse events in intensive care patients

34) Once you gain more expertise in this area, how likely are you to use it in clinical practice?

a. Not at all/unlikely

b. Neutral

c. Very/highly probable
