# Supplementary material for: Prioritizing educational initiatives on emerging technologies for Italian pediatricians: bibliometric review and a survey
Source: Ital J Pediatr. 2023 Sep 5;49:112. doi: 10.1186/s13052-023-01512-w (PMC10478260; doi:10.1186/s13052-023-01512-w)
Supplement: Supplementary file 1 — Supplementary Material 1 [file 13052_2023_1512_MOESM1_ESM.docx]

To the Editor

The Italian Journal Of Pediatrics

Subject: Manuscript Submission - Prioritizing Educational Initiatives on Emerging Technologies for Italian Pediatricians: bibliometric review and a survey

Dear Editor,

I am writing to submit a manuscript entitled "Prioritizing Educational Initiatives on Emerging Technologies for Italian Pediatricians: bibliometric review and a survey” for consideration for publication in the Italian Journal of Pediatrics.

As technological advancements continue to reshape healthcare, it is imperative to explore the integration of emerging technologies in medical specialties, including pediatrics. Our study aimed to address this need by identifying current trends in emerging technologies in pediatrics through a comprehensive bibliometric review and assessing the educational needs and willingness to integrate these technologies among Italian pediatricians via a survey.

Our research employed a multidisciplinary approach, with a working group of pediatricians representing diverse backgrounds leading the study. We focused on key topics, including virtual reality, telehealth, natural language processing, smartphone applications, robotics, genomics, and artificial intelligence. Through a bibliometric analysis, we identified emerging applications within each topic, providing valuable insights into the current landscape of technological advancements in pediatrics.

To complement our bibliometric review, we developed and distributed a detailed online questionnaire to Italian pediatricians. The responses from 1,540 participants, predominantly medical doctors with extensive experience as family pediatricians, allowed us to assess the educational needs and willingness to adopt emerging technologies. The findings highlighted a significant gap between the current and potential usage of these technologies among Italian pediatricians, emphasizing the urgent need for comprehensive education programs to bridge this divide.

Our study makes several noteworthy contributions to the field. Firstly, we have successfully developed a replicable strategy for identifying emerging themes in medical technologies relevant to pediatrics. This strategy can serve as a valuable framework for future research and educational initiatives in integrating emerging technologies in healthcare. Secondly, our findings underscore the importance of addressing gender and age disparities in technology adoption, with recommendations for targeted educational interventions to ensure equitable access and utilization of emerging technologies in pediatrics.

We believe that our manuscript aligns closely with the scope and objectives of the Italian Journal of Pediatrics..

Thank you for considering our manuscript for publication. We confirm that all Authors participated in the development of the work and in its writing and that the results are original and not considered by other scientific journals.

We look forward to your favorable response and the chance to address any queries or provide additional information.

Sincerely,

Alberto Eugenio Tozzi
